# Palladium Nanoparticles-Based Fluorescence Resonance Energy Transfer Aptasensor for Highly Sensitive Detection of Aflatoxin M_1_ in Milk

**DOI:** 10.3390/toxins9100318

**Published:** 2017-10-13

**Authors:** Hui Li, Daibin Yang, Peiwu Li, Qi Zhang, Wen Zhang, Xiaoxia Ding, Jin Mao, Jing Wu

**Affiliations:** 1 Oil Crops Research Institute, Chinese Academy of Agricultural Sciences, Wuhan 430062, China; lihui_whu@whu.edu.cn (H.L.); ydbocriabc@hotmail.com (D.Y.); zhangwen@oilcrops.cn (W.Z.); dingdin2355@sina.com (X.D.); maojin106@whu.edu.cn (J.M.); wujing19900103@sina.com (J.W.); 2 Laboratory of Quality & Safety Risk Assessment for Oilseed Products (Wuhan), Ministry of Agriculture, Wuhan 430062, China; 3 Key Laboratory of Detection for Mycotoxins, Ministry of Agriculture, Wuhan 430062, China; 4 Quality Inspection & Test Center for Oilseed Products, Ministry of Agriculture, Wuhan 430062, China

**Keywords:** aflatoxin M_1_, palladium nanoparticles, aptasensor, fluorescence resonance energy transfer

## Abstract

A highly sensitive aptasensor for aflatoxin M_1_ (AFM_1_) detection was constructed based on fluorescence resonance energy transfer (FRET) between 5-carboxyfluorescein (FAM) and palladium nanoparticles (PdNPs). PdNPs (33 nm) were synthesized through a seed-mediated growth method and exhibited broad and strong absorption in the whole ultraviolet-visible (UV-Vis) range. The strong coordination interaction between nitrogen functional groups of the AFM_1_ aptamer and PdNPs brought FAM and PdNPs in close proximity, which resulted in the fluorescence quenching of FAM to a maximum extent of 95%. The non-specific fluorescence quenching caused by PdNPs towards fluorescein was negligible. After the introduction of AFM_1_ into the FAM-AFM_1_ aptamer-PdNPs FRET system, the AFM_1_ aptamer preferentially combined with AFM_1_ accompanied by conformational change, which greatly weakened the coordination interaction between the AFM_1_ aptamer and PdNPs. Thus, fluorescence recovery of FAM was observed and a linear relationship between the fluorescence recovery and the concentration of AFM_1_ was obtained in the range of 5–150 pg/mL in aqueous buffer with the detection limit of 1.5 pg/mL. AFM_1_ detection was also realized in milk samples with a linear detection range from 6 pg/mL to 150 pg/mL. The highly sensitive FRET aptasensor with simple configuration shows promising prospect in detecting a variety of food contaminants.

## 1. Introduction

Aflatoxins (AFs), which are highly toxic mycotoxins produced by *Aspergillus parasiticus*, *Aspergillus flavus* and *Aspergillus nomius* (rarely), present in a wide range of food and feed commodities [1,2]. The exposure of aflatoxin B_1_ (AFB_1_)-contaminated feed to lactating mammals will lead to the conversion of AFB_1_ into aflatoxin M_1_ (AFM_1_) through hydroxylation under liver cytochrome P450 catalysis [3,4]. AFM_1_, which have intense hepatotoxic and carcinogenic effects and have been designated as group1 carcinogen by the International Agency for Research on Cancer (IARC) of the World Health Organization (WHO) in 2002 [5], will subsequently be secreted in the milk of lactating mammals. As AFM_1_ is quite stable during general pasteurization and storage process and will not be destroyed until the temperature exceeds 299 °C, it will enter human body through milk drinking and endanger human health [6,7]. Therefore, many countries have stipulated the maximum residue limit (MRL) of AFM_1_ in dairy and dairy products. In the European Community, the MRL level for the presence of AFM_1_ in milk has been set at 0.05 ng/mL [8]. According to regulations of the U.S. Food and Drug Administration [9] and the Chinese government [10], the MRL level of AFM_1_ in milk and milk products should not exceed 0.5 ng/mL. Hence it is very important to monitor the concentration of AFM_1_ in dairy products to ensure human health and consumption safety.

In the past few years, various testing methods have been developed for AFM_1_ detection, which includes thin-layer chromatography (TLC) [11,12], enzyme-linked immunosorbent assay (ELISA) [13,14], high-performance liquid chromatography (HPLC) [15,16], liquid chromatography-tandem mass spectrometry (LC-MS) [17,18] and immunosensors [19,20]. Compared with those mentioned above, the aptasensor, which exhibits unique advantages such as being less expensive, easier to operate and suitable for on-site analyses, has attracted increasing attention recently. The aptasensor is referred to as a new category of biosensor constructed with aptamers as recognition elements, which show noticeable advantages over antibodies such as small size, good reproducibility, high stability, easy chemical fabrication with signal moieties and low immunogenicity [21]. Recently, Dinçkaya et al. developed an impedimetric AFM_1_ biosensor based on a 21-mer aptamer sequence for AFM_1_ and gold nanoparticles [22]. Afterwards, electrochemical [23,24] and fluorescent [25] aptasensors have also been developed for AFM_1_ detection. Despite the achievements made in the AFM_1_ aptasensors, it is still a challenge to develop new techniques for AFM_1_ detection with improved sensitivity, simplicity and stability.

Fluorescence resonance energy transfer (FRET), which is a non-radiative energy transfer process occurring between energy donor and acceptor in close proximity (normally 1–10 nm) through long-range dipole-dipole interactions, has been widely used in quantitative determination of biomolecules [26,27], small molecules [28,29] and metal ions [30,31] because of its homogeneous nature and high sensitivity. The fluorescence quenching ability of energy acceptors plays an important role in determining the analytical sensitivity in the FRET-based analysis. Larger palladium nanoparticles (PdNPs ≥ 30 nm), which exhibit broad and strong absorption in the whole ultraviolet-visible (UV-Vis) spectrum range with high molar extinction coefficient [32], have received great attention as an energy acceptor for FRET-based biological analysis application in recent years. Additionally, an ultrasensitive biosensing platform for DNA and protein detection have been developed based on the excellent luminescence quenching ability of PdNPs towards different fluorescent dyes, such as FAM and rhodamine [32]. 

Herein, we combined the excellent luminescence quenching ability of PdNPs towards fluorescent dyes with the highly specific binding ability of the AFM_1_ aptamer towards AFM_1_ to develop a highly sensitive PdNPs-based FRET aptasensor for AFM_1_ detection. When a FAM-labeled AFM_1_ aptamer is incubated with PdNPs, the energy donor was brought close to the energy acceptor in the range from 1 nm to 10 nm through the strong coordination interaction between the nitrogen functional groups of the AFM_1_ aptamer and PdNPs, thus resulting in the fluorescence quenching of FAM. However, after the introduction of AFM_1_ into the FRET quenching system, AFM_1_ aptamer preferentially bound with AFM_1_ accompanied with its conformational change, which greatly weakened the coordination interaction between AFM_1_ aptamer and PdNPs. Thus, the distance between FAM and PdNPs was enlarged and thereby the fluorescence recovery of FAM was observed in a AFM_1_ concentration-dependent manner. The AFM_1_ aptasensor also performed well in milk samples. 

## 2. Results and Discussion

### 2.1. Construction of the FRET Aptasensor for AFM_1_

**Scheme 1:**
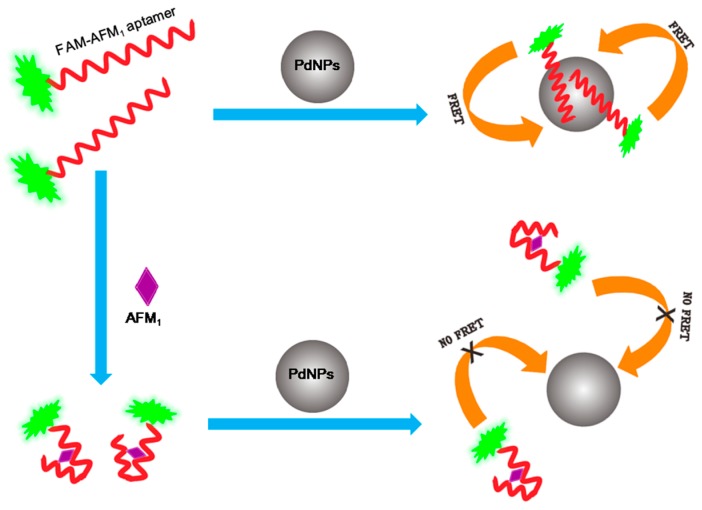
Schematic illustration of the biosensor for aflatoxin M_1_ (AFM_1_) detection based on aptamer-bridged fluorescence resonance energy transfer (FRET) from 5-carboxyfluorescein (FAM) to palladium nanoparticles (PdNPs).

The AFM_1_ aptasensor was constructed based on aptamer-bridged FRET between FAM and PdNPs, as shown in Scheme 1. It has been reported that there was a strong coordination effect between nitrogen functional groups of single-stranded DNA (ssDNA) and PdNPs [32,33]. However, after the hybridization between ssDNA and its complementary chains occurred, the coordination effect was greatly weakened as fewer heteroatoms were exposed to PdNPs in the double helix structure of DNA. In our design, the strong coordination effect between the 5′-FAM-labeled AFM_1_ aptamer and PdNPs brought the fluorescence donor FAM close to the fluorescence acceptor PdNPs, which resulted in the occurrence of FRET, and the fluorescence quenching of FAM was observed. After AFM_1_ was introduced into the FAM-AFM_1_ aptamer-PdNPs FRET system, AFM_1_ aptamer preferentially bound to AFM_1_ accompanied with its conformational change, which largely weakened the coordination effect between the AFM_1_ aptamer and PdNPs. Thus FAM was separated from PdNPs and the FRET process was blocked. Therefore, the fluorescence recovery of FAM was observed and the degree of fluorescence recovery was in a positive AFM_1_ concentration-dependent manner.

### 2.2. Properties Characterization of the Energy Acceptor

**Figure 1:**
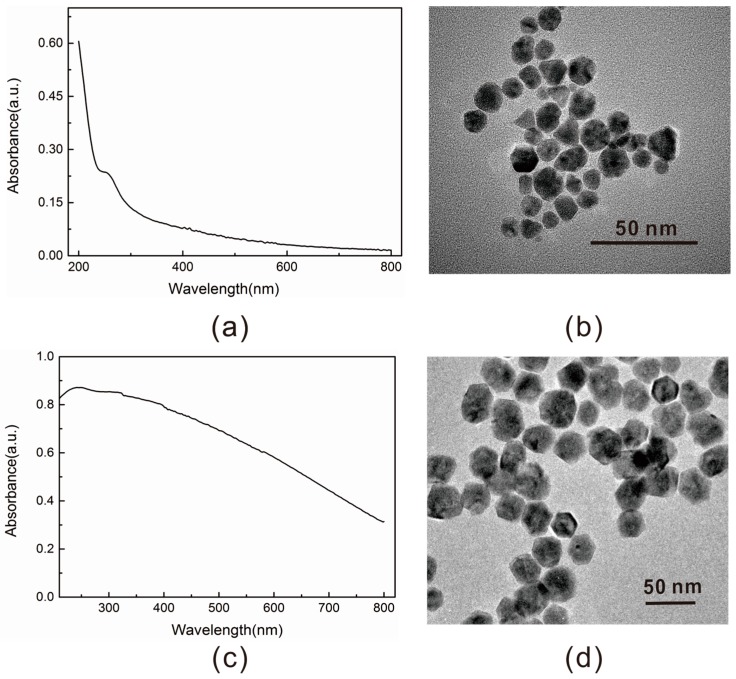
(**a**) Ultraviolet-visible (UV/Vis) absorption spectra of the 12 nm PdNPs; (**b**) transmission electron microscope (TEM) images of the 12 nm PdNPs; (**c**) UV/Vis absorption spectra of the 33 nm PdNPs; (**d**) TEM images of the 33 nm PdNPs.

As the 5′-FAM-labeled AFM_1_ aptamer was negatively charged, sodium citrate-modified PdNPs with negative charge were used in this biosensor to avoid the side effect caused by electrostatic attraction which would also bring FAM close to PdNPs. Firstly, 12 nm Pd seeds (Figure 1b) whose light absorption was centered in the ultraviolet region and was very weak in the visible range (Figure 1a) were synthesized using a sodium ascorbate reduction method. Then, larger PdNPs with an average diameter of 33 nm (Figure 1d) were synthesized on the basis of the 12 nm Pd seeds according to a seed-mediated growth method. The UV-Vis absorption spectrum of the 33 nm PdNPs in Figure 1c clearly showed that it exhibited strong absorption in nearly the whole UV-Vis spectral range, which overlaps well with the emission spectrum of FAM, which was essential for FRET occurrence between FAM and PdNPs. 

### 2.3. Construction of the AFM_1_ Aptasensor

**Figure 2:**
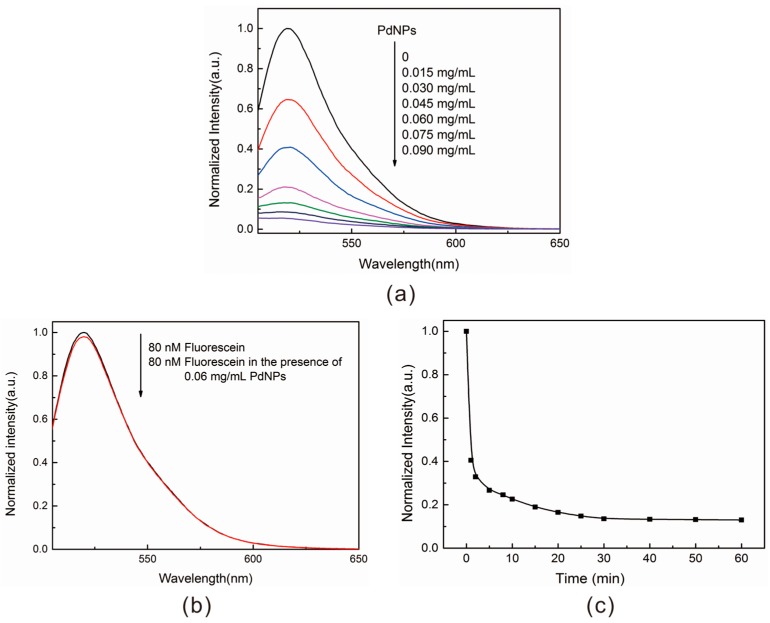
(**a**) Fluorescence quenching of 5′-FAM-labeled AFM_1_ aptamer after incubated with different concentrations of 33 nm PdNPs (0, 0.015 mg/mL, 0.030 mg/mL, 0.045 mg/mL, 0.060 mg/mL, 0.075 mg/mL, 0.090 mg/mL). (**b**) Fluorescence quenching of fluorescein in the presence of 0.060 mg/mL PdNPs (33 nm). (**c**) Time dependence of the fluorescence quenching degree for 80 nM 5′-FAM-labeled AFM_1_ aptamer caused by 0.060 mg/mL PdNPs (33 nm). All experiments were performed in HEPES buffer under excitation at 480 nm and emission at 520 nm.

To investigate the energy transfer efficiency between the FAM donor and PdNPs acceptor pair, an increasing concentration of 33 nm PdNPs were added into a fixed amount of 5′-FAM-labeled AFM_1_ aptamer (80 nM). After incubation in HEPES buffer (20 mM, pH = 7.0) containing 5 mM KCl and 5 mM MgCl_2_ for a short while, a PdNPs concentration-dependent fluorescence quenching phenomenon of 5′-FAM-labeled AFM_1_ aptamer was observed with the maximum quenching efficiency reaching 95%, as indicated in Figure 2a. In order to investigate the non-specific fluorescence quenching caused by PdNPs towards fluorescein dye, fluorescein dye at a final concentration of 80 nM was mixed with PdNPs (0.060 mg/mL) in HEPES buffer for 1 h and then the fluorescence intensity of fluorescein was measured. From Figure 2b it clearly indicated that the non-specific fluorescence quenching caused by PdNPs towards fluorescein could be eliminated. Therefore, the effective fluorescence quenching of FAM caused by PdNPs was ascribed to the strong coordination effect between nitrogen functional groups of the AFM_1_ aptamer and PdNPs, which brought FAM close to PdNPs resulting the occurrence of FRET. The time dependence of fluorescence quenching efficiency indicated in Figure 2c suggested that it only took 30 min to reach the quenching equilibrium. In the following fluorescence recovery experiments, In order to ensure reaching the quenching equilibrium and obtain stable fluorescence signal, 1 h incubation time was chosen for the fluorescence quenching experiment. 

### 2.4. AFM_1_ Detection in Aqueous Buffer Solution

**Figure 3:**
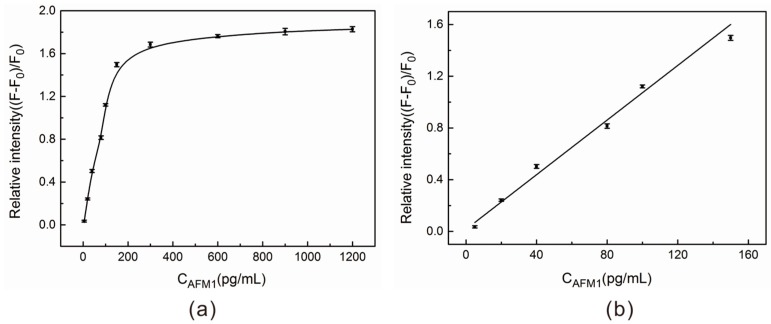
(**a**) The fluorescence recovery trend line in accordance with different concentrations of AFM_1_ (5, 20, 40, 80, 100, 150, 300, 600, 900, 1200 pg/mL). *F*_0_ represents the fluorescence intensity in the absence of AFM_1_. (**b**) The linear relationship between the fluorescence recovery degree (at 520 nm) and the concentration of AFM_1_ within the range from 5 pg/mL to 150 pg/mL, data were presented as average ± SD from three independent measurements. Experiments were conducted in the presence of 80 nM 5′-FAM-labeled AFM_1_ aptamer and 0.060 mg/mL PdNPs in HEPES buffer under excitation at 480 nm.

**Figure 4:**
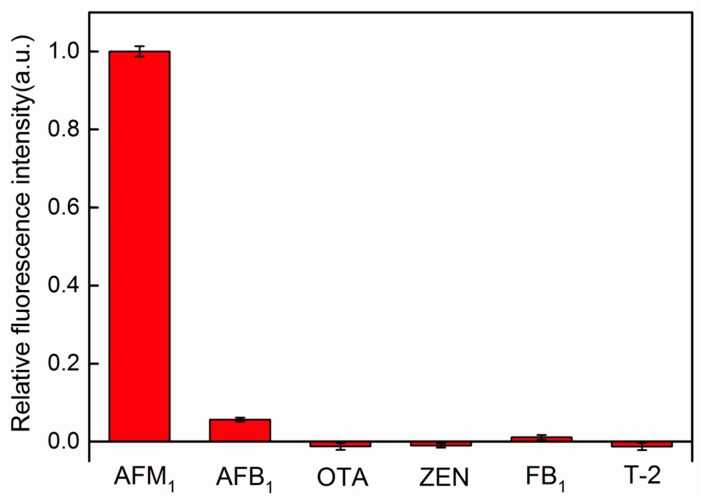
Relative fluorescence intensity ((*F*_other mycotoxins_ − *F*_0_)/(*F*_AFM1_ − *F*_0_)) of the aptamer-bridged FRET biosensor for AFM_1_ detection in the presence of different mycotoxins, where *F*_0_ is the fluorescence intensity in the absence of AFM_1_ or other mycotoxins. Data were presented as average ±SD from three independent measurements. The concentration of mycotoxins were all 150 pg/mL. Experiments were conducted in HEPES buffer under excitation at 480 nm.

As illustrated in Scheme 1, after AFM_1_ was introduced into the 5′-FAM-AFM_1_ aptamer-PdNPs FRET system in the HEPES buffer, the AFM_1_ aptamer preferentially bound to AFM_1_ accompanied by its conformational change, which greatly weakened the coordination effect between the AFM_1_ aptamer and PdNPs. Therefore, FAM was separated from PdNPs and the FRET process was inhibited. Meanwhile, the fluorescence of FAM was restored and the degree of fluorescence recovery was in an AFM_1_ concentration-dependent manner, as indicated in Figure 3a. A linear relationship between the fluorescence recovery of FAM and the concentration of AFM_1_ in the range from 5 pg/mL to 150 pg/mL was obtained in the HEPES buffer, with the detection limit of 1.5 pg/mL (calculated as the concentration corresponding to three times of the standard deviation of the background signal from seven independent measurements) (Figure 3b). Compared to the previously reported structure, switching aptamer-based FRET assay for AFM_1_ detection with a linear detection range from 25 ng/kg to 2000 ng/kg [25], and the time-resolved fluorescent competitive immunochromatographic assay for AFM_1_ detection in milk with a linear dynamic range of 0.1–2.0 ng/mL [34], the sensitivity of the present sensor is significantly improved, which shows great potential to detect lower concentration of AFM_1_ in milk samples. The performance improvement of this FRET largely relied on the excellent fluorescence quenching ability of PdNPs towards FAM, with almost negligible non-specific fluorescence quenching. Other interfering toxins, including AFB_1_, OTA, ZEN, FB_1_ and *T*-2 toxin, were added individually into the FAM-AFM_1_ aptamer-PdNPs FRET system in the place of AFM_1_ under the same experimental procedures to examine the specificity of this FRET biosensor for AFM_1_. It can be seen from Figure 4 that the interference toxins all cause negligible fluorescence variation of FAM compared to AFM_1_, which firmly indicated the excellent specificity of this developed FRET biosensor towards AFM_1_ as a result of the high binding affinity between AFM_1_ aptamer and AFM_1_.

### 2.5. AFM_1_ Detection in Milk Samples

**Figure 5:**
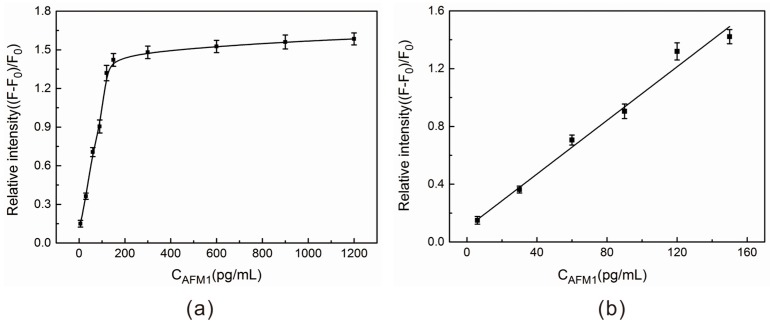
(**a**) Fluorescence recovery of the biosensor with the introduction of different amounts of AFM_1_ (6, 30, 60, 90, 120, 150, 300, 600, 900, 1200 pg/mL) in 100-diluted milk sample. (**b**) The linear relationship between the fluorescence recovery (at 520 nm) and the concentration of AFM_1_ within the range of 6–150 pg/mL in diluted milk, data were presented as average ±SD from three independent measurements. All experiments were performed in the presence of 80 nM 5′-FAM-labeled AFM_1_ aptamer and 0.060 mg/mL PdNPs under excitation at 480 nm.

In order to ensure consumption safety and human health, it is very important to monitor the concentration of AFM_1_ in milk. In this paper, AFM_1_ detection was also realized in 100-fold diluted milk sample with HEPES buffer under the same experimental procedures as that in the aqueous buffer solution. It can be seen from Figure 5a that the fluorescence of FAM was restored in a AFM_1_ concentration-dependent manner. And the degree of fluorescence restoration was linear related to the concentration of AFM_1_ in the range from 6 pg/mL to 150 pg/mL, with a detection limit of 1.8 pg/mL (calculated as the concentration corresponding to three times of the standard deviation of the background signal from seven independent measurements) (Figure 5b). The relatively narrower linear range and higher detection limit may be ascribed to the complexity of the milk sample. Standard addition experiments were conducted to examine the feasibility of this AFM_1_ biosensor in practical AFM_1_-free milk samples. The satisfactory recoveries from 92% to 106.5% in Table 1 convincingly demonstrates that this FRET biosensor based on the efficient fluorescence resonance energy transfer between FAM and PdNPs has great potential in practical application. 

## 3. Conclusions

In summary, a highly sensitive FRET aptasensor for AFM_1_ detection has been constructed based on the excellent fluorescence quenching ability of PdNPs towards FAM with negligible non-specific fluorescence quenching. The application of AFM_1_ aptamer with high affinity and specificity towards AFM_1_ also contributes to the good performance of this biosensor in both aqueous buffer solution and milk samples. In consideration of its simple configuration and operation, the homogeneous FRET aptasensor can be widely used to detect a variety of food contaminants, such as other biotoxins in the future.

## 4. Materials and Methods 

### 4.1. Materials

Standard solutions of AFM_1_, AFB_1_, ochratoxin A (OTA), zearalenone (ZEN), fumonisin B_1_ (FB_1_) and *T*-2 toxin were purchased from Sigma-Aldrich (St. Louis, MO, USA). 5′-FAM-labeled AFM_1_ aptamer (5′-FAM-ACTGCTAGAGATTTTCCACAT-3′) was provided by Sangon Biotechnology Co., Ltd. (Shanghai, China). The other reagents were all from Sinopharm Chemical Reagent Co., Ltd. (Shanghai, China). The solvents and reagents were used as received without further purification. All aqueous solutions were prepared in ultrapure water obtained from a Milli-Q purification system (Millipore, Kankakee, IL, USA).

### 4.2. Instrumentation

The size and morphology of palladium seeds and larger PdNPs were characterized by a FEI Tecnai G2 F30 transmission electron microscope with an acceleration voltage of 200 kV. The UV-vis absorption measurements were conducted on a Thermo-Spectronic Unicam UV500 spectrometer (Thermo Spectronic, Waltham, MA, USA). The fluorescence spectra were recorded on a HITACHI F-4500 fluorescence spectrometer (HITACHI, Tokyo, Japan). 

### 4.3. Synthesis of Sodium Citrate Capped Palladium Seeds

Palladium seeds were synthesized according to a reported procedure [35]. 20% freshly prepared aqueous solutions of sodium citrate (100 µL) and 1% Na_2_PdCl_4_ in water (735 µL) were both added into 47 mL ultrapure water. After the solution was heated to boiling, 0.1% hot sodium ascorbate (2.5 mL) was introduced quickly into the mixture. Boiling under reflux was continued for another 30 min. Then the solution was cooled down to room temperature naturally and filtered through a 0.22 um Millipore membrane filter.

### 4.4. The Synthesis of 33 nm PdNPs

The synthesis of 33 nm PdNPs was accomplished by a seed-mediated growth method reported by Lu et al. [36]. 10 mL aqueous solution of H_2_PdCl_4_ with a concentration of 1 mM was placed in a 50 mL round-bottom flask. And 3 mL of the synthesized palladium seeds were added. Then an excess amount of aqueous solution of ascorbic acid (100 mM, 1.2 mL) was introduced into the above solutions under extensive stirring. The color of the solution readily changed from pale yellow to a dark brown color, which suggested the formation of larger PdNPs. The resultant solution was stirred for another 5 min at room temperature. Next, the obtained PdNPs were centrifuged and washed with ultrapure water for three times. Finally, the products were redispersed in 3 mL of ultrapure water for further use.

### 4.5. Quenching Measurements

The concentration of PdNPs used in the FRET system were optimized against a fixed concentration of 5′-FAM-labeled AFM_1_ aptamer, that is, 80 nM. For optimization, the concentrations of PdNPs were set at 0, 0.015 mg/mL, 0.030 mg/mL, 0.045 mg/mL, 0.060 mg/mL, 0.075 mg/mL, 0.090 mg/mL. They were incubated in HEPES buffer (20 mM, pH = 7.0) containing 5 mM KCl and 5 mM MgCl_2_ for 1 h and then the fluorescence intensities were recorded under excitation at 480 nm and emission at 520 nm. The time-dependent fluorescence intensities were obtained by incubating a fixed concentration of 5′-FAM-labeled AFM1 aptamer (80 nM) with PdNPs in a concentration of 0.060 mg/mL from 1 min to 60 min. 

### 4.6. AFM_1_ Detection in Aqueous Buffer Solution

In a typical FRET analysis process, various concentrations of AFM_1_ (0, 5 pg/mL, 20 pg/mL, 40 pg/mL, 80 pg/mL, 100 pg/mL, 150 pg/mL, 300 pg/mL, 600 pg/mL, 900 pg/mL, 1200 pg/mL) were first mixed with the 5′-FAM-labeled AFM_1_ aptamer (80 nM) in HEPES buffer, respectively, and the mixtures were all incubated at room temperature for 2 h. Afterwards PdNPs was added individually into the above mixtures with an ultimate concentration of 0.060 mg/mL, followed by incubation for another 1 h at room temperature. Finally, the fluorescence intensity of the reaction mixture was recorded under excitation at 480 nm and emission at 520 nm. To examine the specificity of the FRET aptasensor, a list of other mycotoxins including AFB_1_, OTA, ZEN, FB_1_ and *T*-2 toxin were added into the FAM-AFM_1_ aptamer-PdNPs FRET system in place of AFM_1_ following the same experimental procedures.

### 4.7. AFM_1_ Detection in Milk Samples

The milk samples (7% fat content) were purchased from the local market of Wuhan, China. They were first centrifuged at 5000 rpm for 10 min at 25 °C to remove the fat and the supernatant were collected respectively. For the determination of AFM_1_ in milk samples, the supernatant was 100-fold diluted with HEPES buffer without further processing, and the same assay procedure as in the HEPES buffer solution was followed. Standard addition method was adopted to determine the concentration of AFM_1_ in AFM_1_-free milk samples. 

## Figures and Tables

**Table 1:** Determination of AFM_1_ in three milk samples.

Sample No.	Added (pg/mL)	Found (pg/mL) ^a^	Recovery (%)	Relative Standard Deviation (RSD) (%) *n* = 3
1	10	9.20	92.0	3.4
2	70	65.64	93.8	4.8
3	120	127.85	106.5	4.9

^a^ Mean value of three determinations by the aptasensor.

## References

[1] Torres A.M., Barros G.G., Palacios S.A., Chulze S.N., Battilani P. (2014). Review on pre- and post-harvest management of peanuts to minimize aflatoxin contamination. Food Res. Int..

[2] Bakirci I. (2001). A study on the occurrence of aflatoxin M_1_ in milk and milk products produced in Van province of Turkey. Food Control.

[3] Richard J.L. (2007). Some major mycotoxins and their mycotoxicoses-an overview. Int. J. Food Microbiol..

[4] Kanungo L., Pal S., Bhand S. (2011). Miniaturised hybrid immunoassay for high sensitivity analysis of aflatoxin M_1_ in milk. Biosens. Bioelectron..

[5] International Agency for Research on Cancer (2002). Monograph on the Evaluation of Carcinogenic Risks in Humans: Some Traditional Herbal Medicines, Some Mycotoxins.

[6] Iha M.H., Barbosa C.B., Okada I.A., Trucksess M.W. (2013). Aflatoxin M_1_ in milk and distribution and stability of aflatoxin M_1_ during production and storage of yoghurt and cheese. Food Control.

[7] Badea M., Micheli L., Messia M.C., Candigliota T., Marconi E., Mottram T., Velasco-Garcia M., Moscone D., Palleschi G. (2004). Aflatoxin M_1_ determination in raw milk using a flow-injection immunoassay system. Anal. Chim. Acta.

[8] (2006). European Commission, Commission Regulation (EC) No 1881/2006 of 19 December 2006 setting maximum levels for certain contaminants in foodstuffs. Off. J. Eur. Union.

[9] US Food and Drug Administration (2005). 400 Whole Milk, Low Fat Milk, Skim Milk-Aflatoxin M1 (cpg 7106.210), FDA Compliance Policy Guides.

[10] MoH (Ministry of Health) P.R. China (2011). Maximum Residue Level of Mycotoxin in Food-National Regulations for Food Safety.

[11] Lin L.M., Zhang J., Wang P., Wang Y.S., Chen J.P. (1998). Thin-layer chromatography of mycotoxins and comparison with other chromatographic methods. J. Chromatogr. A.

[12] Filazi A., Ince S., Temamogullari F. (2010). Survey of the occurrence of aflatoxin M_1_ in cheeses produced by dairy ewe’s milk in Urfa city, Turkey. Ankara Üniv. Vet. Fak. Derg..

[13] Anfossi L., Calderara M., Baggiani C., Giovannoli C., Arletti E., Giraudi G. (2008). Development and application of solvent-free extraction for the detection of aflatoxin M_1_ in dairy products by enzyme immunoassay. J. Agric. Food Chem..

[14] Chadseesuwan U., Sangdokmai A., Pimpitak U., Puthong S., Palaga T., Komolpis K. (2016). Production of a monoclonal antibody against aflatoxin M_1_ and its application for detection of aflatoxin M_1_ in fortified milk. J. Food Drug Anal..

[15] Shuib N.S., Makahleh A., Salhimi S.M., Saad B. (2017). Determination of aflatoxin M_1_ in milk and dairy products using high performance liquid chromatography-fluorescence with post column photochemical derivatization. J. Chromatogr. A.

[16] Hashemi M., Taherimaslak Z. (2014). Determination of aflatoxin M-1 in liquid milk using high performance liquid chromatography with fluorescence detection after magnetic solid phase extraction. RSC Adv..

[17] Huang S.M., Hu D., Wang Y., Zhu F., Jiang R.F., Ouyang G.F. (2015). Automated hollow-fiber liquid-phase microextraction coupled with liquid chromatography/tandem mass spectrometry for the analysis of alfatoxin M-1 in milk. J. Chromatogr. A.

[18] Campone L., Piccinelli A.L., Celano R., Russo M., Rastrelli L. (2013). Rapid analysis of aflatoxin M-1 in milk using dispersive liquid-liquid microextraction coupled with ultrahigh pressure liquid chromatography tandem mass spectrometry. Anal. Bioanal. Chem..

[19] Parker C.O., Lanyon Y.H., Manning M., Arrigan D.W.M., Tothill I.E. (2009). Electrochemical immunochip sensor for aflatoxin M_1_ detection. Anal. Chem..

[20] Gan N., Zhou J., Xiong P., Hu F.T., Cao Y.T., Li T.H., Jiang Q.L. (2013). An ultrasensitive electrochemiluminescent immunoassay for aflatoxin M_1_ in milk, based on extraction by magnetic graphene and detection by antibody-labeled CdTe quantum dots-carbon nanotubes nanocomposite. Toxins.

[21] Chen A.L., Yang S.M. (2015). Replacing antibodies with aptamers in lateral flow immunoassay. Biosens. Bioelectron..

[22] Dinçkaya E., Kınık Ö., Sezgintürk M.K., Altuğ Ҫ., Akkoca A. (2011). Development of an impedimetric aflatoxin M_1_ biosensor based on a DNA probe and gold nanoparticles. Biosens. Bioelectron..

[23] Nguyen B.H., Tran L.D., Do Q.P., Nguyen H.L., Tran N.H., Nguyen P.X. (2013). Label-free detection of aflatoxin M_1_ with electrochemical Fe_3_O_4_/polyaniline-based aptasensor. Mater. Sci. Eng. C.

[24] Liu J.L., Zhao M., Zhuo Y., Chai Y.Q., Yuan R. (2017). Highly efficient intramolecular electrochemiluminescence energy transfer for ultrasensitive bioanalysis of alfatoxin M_1_. Chem. Eur. J..

[25] Sharma A., Catanante G., Hayat A., Istamboulie G., Rejeb I.B., Bhand S., Marty J.L. (2016). Development of structure switching aptamer assay for detection of aflatoxin M_1_ in milk sample. Talanta.

[26] Wegner K.D., Lindén S., Jin Z., Jennings T.L., Khoulati R., van Bergen en Henegouwen P.M.P., Hildebrandt N. (2014). Nanobodies and nanocrystals: Highly sensitive quantum dot-based homogeneous FRET immunoassay for serum-based EGFR detection. Small.

[27] Lao Y.H., Chi C.W., Friedrich S.M., Peck K., Wang T.H., Leong K.W., Chen L.C. (2016). Signal-on protein detection via dye translocation between aptamer and quantum dot. ACS Appl. Mater. Interfaces.

[28] Wang Y.H., Jiang K., Zhu J.L., Zhang L., Lin H.W. (2015). A FRET-based carbon dot-MnO_2_ nanosheet architecture for glutathione sensing in human whole blood samples. Chem. Commun..

[29] Arola H.O., Tullila A., Kiljunen H., Campbell K., Siitari H., Nevanen T.K. (2016). Specific noncompetitive immunoassay for HT-2 mycotoxin detection. Anal. Chem..

[30] Chen G.Z., Jin Y., Wang L., Deng J., Zhang C.X. (2011). Gold nanorods-based FRET assay for ultrasensitive detection of Hg^2+^. Chem. Commun..

[31] Maity D., Karthigeyan D., Kundu T.K., Govindaraju T. (2013). FRET-based rational strategy for ratiometric detetction of Cu^2+^ and live cell imaging. Sensor Actuators B Chem..

[32] Li H., Sun D.E., Liu Z.H. (2015). Ultrasensitive biosensing platform based on the luminescence quenching ability of plasmonic palladium nanoparticles. Chem. Eur. J..

[33] Li H., Shi L., Sun D.E., Li P.W., Liu Z.H. (2016). Fluorescence resonance energy transfer biosensor between upconverting nanoparticles and palladium nanoparticles for ultrasensitive CEA detection. Biosens. Bioelectron..

[34] Tang X.Q., Zhang Z.W., Li P.W., Zhang Q., Jiang J., Wang D., Lei J.W. (2015). Sample-pretreatment-free based high sensitive determination of aflatoxin M_1_ in raw milk using a time-resolved fluorescent competitive immunochromatographic assay. RSC Adv..

[35] Meyer D.A., Albrecht R.M. (2003). Sodium ascorbate method for the synthesis of colloidal palladium particles of different sizes. Microsc. Microanal..

[36] Lu L.H., Wang H.S., Xi S.Q., Zhang H.J. (2002). Improved size control of large palladium nanoparticles by a seeding growth method. J. Mater. Chem..

